# Editorial: The Role of Complement in Microbial Infections

**DOI:** 10.3389/fimmu.2021.640092

**Published:** 2021-03-04

**Authors:** Heribert Stoiber, Nicole Thielens, Reinhard Würzner, Iara De Messias Reason

**Affiliations:** ^1^Institute of Virology, Innsbruck Medical University, Innsbruck, Austria; ^2^Université Grenoble Alpes, CNRS, CEA, IBS, Grenoble, France; ^3^Institute of Hygiene and Medical Microbiology, Innsbruck Medical University, Innsbruck, Austria; ^4^Laboratório de Imunopatologia Molecular, Universidade Federal do Paraná, Curitiba, Brazil

**Keywords:** complement, pathogens, bacteria, viruses, fungi, parasites

Complement plays a major role in immune defense against all different types of pathogens, namely bacteria, viruses, fungi, and parasites. All these pathogens try to evade complement attack by numerous mechanisms. The 14 articles of this themed collection highlight the latest advances regarding this complement-pathogen relationship. A theme, which is found frequently is the binding of complement regulator proteins on the surface of pathogens to avoid complement-mediated destruction. A major regulator is hereby factor H (fH), which is recruited from fluid phase. The group of Marc Thilo Figge provided a sophisticated mathematical model, which allows quantifying fH mediated discrimination to self vs. non-self in fluid phase and steady state (Tille et al.). This dynamic model may be used to not only define critical fH concentrations on the surface of pathogens, but also to model interactions of fH mutants and their effect under pathophysiological conditions (Tille et al.).

Kristensen et al. from Copenhagen, Denmark show in a prospective, observational study that different complement factors and complement activation products could be potential prognostic markers for necrotizing soft-tissue infection (NSTI), a life-threatening condition whose early diagnosis is mandatory for successful outcome. The authors found that both complement lectin and classical pathway were depleted during NSTI due to complement activation and that complement could be a good predictor of mortality in patients with NSTI, being a promising novel prognostic tool for the disease (Kristensen et al.).

Three contributions focus on bacteria. The first by Röttgerding and Kraiczy from Frankfurt, Germany, is summarizing immune evasion strategies of relapsing fever spirochetes. Relapsing fever, a neglected arthropod-borne disease, is caused by a number of diverse human pathogenic *Borrelia* species which all inactivate complement by recruiting distinct regulatory proteins, e.g., C1 esterase inhibitor (C1-INH), C4b-binding protein (C4BP), factor H (FH), FH-like protein-1 (FHL-1), and factor H-related proteins FHR-1 and FHR-2, or by binding to individual complement components (Röttgerding and Kraiczy).

The second paper by the group of Lourdes Isaac, Sao Paolo, Brazil, is focusing on the role of properdin in immune defense against *Leptospira biflexa*. Properdin (P) is a positive regulatory protein of the complement alternative pathway (AP), recently been implicated to directly bind to the surface of certain pathogens regardless of the presence of C3bBb (Martinez et al.). In that paper the authors demonstrate direct binding of P to saprophytic *Leptospira*. Most importantly, this microorganism survives in P-depleted human serum while the addition of purified P to P-depleted human serum decreases the number of viable leptospires (Martinez et al.).

The third paper from the group of Jeane Eliete Laguila Visentainer from Maringa, Brazil assesses the association of MBL2 exon 1 polymorphisms with multibacillary leprosy (Tiyo et al.). Mannose-binding lectin (MBL), a recognition protein of the lectin pathway, plays a major role in innate anti-microbial immunity. Different variants on exon 1 of the gene coding for MBL (*MBL2*) influence MBL serum level. In a case-control study including 350 leprosy patients from Southern Brazil, the authors found an association of *MBL2* B variant with a higher risk for development of multibacillary (MB) leprosy. In addition, the TGG and CAG haplotypes presented an increased susceptibility to develop leprosy in general and MB disease, in women, which was not observed in the male population (Tiyo et al.).

Four contributions deal with viruses. The first two by the group of Posch et al., Innsbruck, Austria; in Bermejo-Jambrina et al. they investigated the effect of complement opsonization of HIV-1, which has been shown to enhance infection of dendritic cells (DCs) while enhancing anti-viral immune response. Here they further deciphered the underlying mechanisms by studying the individual role of complement receptors CR3 and CR4. THP-1 cells knocked-out for CD11b (CR3) and CD11c (CR4) were generated using the CRISPR-Cas9 technology and differentiated into THP1-DCs. Evidence is provided for distinct and opposite roles of CR3 and CR4, the latter playing a major role in the inflammatory and anti-viral responses triggered by complement-opsonized HIV (Bermejo-Jambrina et al.). The role of complement receptors was summarized in an additional contribution of this group (Posch et al.) focusing on HIV and DCs. This comprehensive review discussed recent finding and gave a summary of the high potential of DCs primed with complement-opsonized HIV to initiates efficient antiviral and cytotoxic anti-HIV immunity (Posch et al.).

The second review, which focuses on complement-virus interactions was provided by the group of John Bernet Johnson, Thiruvananthapuram, India and addressed the interaction of complement with RNA viruses from a more general point of view (Kumar et al.). The authors discussed all aspects of complement RNA virus interactions from complement activation, to interactions of opsonized RNA viruses with complement receptors and mechanisms to escape from complement-mediated lysis (Kumar et al.).

Malekshahi et al. from Innsbruck, Austria provided evidence that the envelope (E) protein of Zika virus directly bind to proteins of the terminal pathway of complement and interferes with the formation of the membrane-attack complex (MAC). The authors showed that both NS1 and the E proteins work synergistically to protect the virus by limiting MAC formation and thus reducing complement-mediated damage (Malekshahi et al.).

Two contributions focus on fungi. Deshmukh et al. from Innsbruck, Austria, investigated the interaction of Aspergillus-derived galactosaminogalactan (GAG) with platelets and the subsequent complement activation. This effect was exclusively induced by GAG from A. fumigatus and not observed with other Aspergillus species or several Mucorales tested. The induction of C3a and C5a, pro-inflammatory cytokines and the detection of C5b-C9 complexes on platelets might contribute to the reduced number of these cells and this might provide an explanation for the excessive inflammation, thrombocytopenia, and thrombosis observed in infected patients (Deshmukh et al.). The group of Cornelia Speth, Innsbruck, Austria added a further paper to the Research Topic focusing on new aspects on the interaction of Candida and complement (Harpf et al.). Beside reviewing several escape mechanisms from the activated complement system by *C. albicans*, recent findings on the interplay between non-albicans C.-species such as *C. auris* were highlighted (Harpf et al.).

Parasites were the final group of pathogens, which were addressed by three contributions. The group of Jörg Köhl, Lübeck, Germany, investigated the role of C5a/C5aR1 axis activation for early immune responses in a mouse model of *Toxoplasma gondii* (*T. gondii*) infection. *C5ar1–/–* mice suffered from more severe disease progression, which was associated with reduced concentrations of IL-12, IL-27, and IFN-γ in serum (Briukhovetska et al.). This resulted not only in decreased frequency of IFN-γ+ NK cells in the spleen, but also reduced IL12 production by splenic DCs, which is important for IFN-γ production and subsequent iNOS expression in the brain to control acute *T. gondii* infection (Briukhovetska et al.).

Among the virulence factors of *Trypanosoma cruzi* (*T. cruzi*) with a capacity to inhibit complement activation is T. cruzi calreticulin (TcCalr). Ramirez-Toloza et al., Santiago, Chile, and colleagues reviewed the mechanisms by which TcCalr blocks either, the classical or lectin pathway. In addition, they addressed recent findings indicating that TcCalr inhibits angiogenesis, promotes wound healing and reduces tumor growth. As other parasites express Calr, too, the detailed understanding of the role of this virulence factor may provide new strategies to develop anti-parasite therapies and prophylaxis (Ramirez-Toloza et al.).

The manuscript presented by Shao et al. adds a piece of interesting data on the modulation of complement activation by a helminth parasite. The authors showed that a calreticulin protein secreted by *Trichinella spiralis*, TsCRT, interacts with human complement C1q through its S-domain inhibiting C1q-mediated complement activation (1). Furthermore, TsCRT-S was able to inhibit neutrophil activation as well as formation of ROS and neutrophil NETs *in vitro*. The authors suggest a potential therapeutic effect of TsCRT-S in the treatment of C1q/immune complex-related autoimmune and inflammatory diseases (1).

In summary, all published articles provide new exciting data of complement-pathogen interactions and help to elucidate additional pieces of the fascinating jigsaw.

## Author Contributions

All authors listed have made a substantial, direct and intellectual contribution to the work, and approved it for publication.

## Conflict of Interest

The authors declare that the research was conducted in the absence of any commercial or financial relationships that could be construed as a potential conflict of interest.
